# Notes and News

**Published:** 1903-01-10

**Authors:** 


### NOTES AND NEWS.

MISS INA LOCKHEAD MCNEILL has been appointed surgeon to the out-patients at the Wolverhampton Women's Hospital.

THERE is a slight increase in the number of cholera cases in Alexandria. Cairo continues free. The news from the Philippines is bad, as the form of the disease is very severe.

THE sanatorium recently opened at Baguley by the Withington District Council is intended for cases of infectious disease, and not, as previously stated, for cases of consumption.

MR. G. W. PALMER, who gave £2,000 to enable the laboratory of the University of London, South Kensington, to be fitted up, has given a further £1,000 towards its permanent endowment.

IT is announced that the plague is raging in Mexico. It is believed that rats are responsible for the introduction of the disease. Temporary Government hospitals have been opened at Mazatlan, the seat of the outbreak. Mazatlan is a seaport town trading largely with the United States.

MR. J. PEMBERTON has given a site for a children's hospital in connection with the Sunderland Infirmary, and it is hoped to collect £10,000 for the establishment of the institution. There is not sufficient accommodation for children at the infirmary, and the management consider that it is disadvantageous to have children in a general hospital.

SIR HENRY BURDETT, the hon. treasurer of the League of Mercy, has recently been giving addresses on the work of hospitals in London accompanied by views of hospital life. Many inquiries having been made at the offices, 29 Southampton Street, Strand, we are asked to say that the following fixtures have already been made for January :—The Town Hall, Hackney, Thursday, January 15th, at 8 o'clock ; the Town Hall, Camberwell, Thursday, January 22nd, at 8 o'clock ; the Large Hall, Paddington Baths, Paddington, Thursday, January 29th, at 8.30 o'clock.

A GOOD many medical men are amongst those receiving Durbar honours. Surgeon-General William R. Hooper, late of the Indian Medical Service, has been created K.C.S.I., and Surgeon-General Benjamin Franklin, Director-General of the Indian Medical Service, and Sanitary Commissioner with the Government of India, has been made a K.C.I.E. Other honours bestowed are knighthood, companionship of various orders, and medals.

MESSRS. William and Arthur James have placed the Frank James Memorial Home at Cowes at the disposal of Princess Henry of Battenberg to be utilised as a hospital for Cowes, and as a home for district nurses. The conditions are that it shall remain as a memorial to Mr. Frank James, that for the first two years the Messrs. James will provide a subscription of £300 per annum, and after that period, if they are satisfied with the success of the hospital, they will make a free gift of the institution and endow it with £10,000.

UNDER the title of "The Skeleton of the Feast," the *Westminster Review* contains an article drawing attention once again to the well-worn theme of the increase of insanity. The writer does well in pointing out, as has been done scores of times before, that by the early discharge of patients said to be "cured," but of course carrying within themselves the full burden of their hereditary taint, the propagation of this taint is encouraged. The force of his remarks is, however, considerably discounted by his failing to differentiate between registered and unregistered insanity, and by his devoting so large a space to girding at the Lunacy Commissioners for drawing a distinction—a distinction the bearing of which he seems not to grasp—between "real" and "apparent" increase in lunacy. Still anything which draws public attention to the facts is of use, although the remedy proposed, namely, an international conference, would seem to be of all things the most futile.

OUR honourable contemporary *The Globe* newspaper celebrated its centenary on New Year's Day. The issue of that date contains an interesting history of the establishment, politics, proceedings, and vicissitudes of the paper. It owes its foundation to the action of the *Morning Post*, which paper, about a year before the birth of *The Globe*, denied its columns to publishers' advertisements. *The Globe* was practically founded to support the book trade, and started with the title of *The Globe, or Literary Advertiser*. The partnership appears, however, to have been soon dissolved, and the advertisement columns of *The Globe* enhanced the fortunes of new publications as little as did those of the *Morning Post*. The paper passed through the hands of various proprietors and editors during its career. It started at a cost of 6d. per copy and a circulation of about 200 per week. Its politics commenced by being of a non-committal order, changed to a more violent character in the Radical interest, and finally, in 1866, settled down to the good solid Tory lines of to-day. We cannot do better than wish it a continuance of its present success and a long life of usefulness.

